# Influence of homogenization and pasteurization on the physical characteristics, antioxidant properties, and microbial content of VD20 rice milk

**DOI:** 10.1038/s41598-025-88436-z

**Published:** 2025-03-20

**Authors:** Ngoc Kim Giang Tu, Thi Kim Loan Le, Thi Yen Nhi Tran, Long Giang Bach, Tan Phat Dao

**Affiliations:** 1 Faculty of Chemical Engineering And Food Technology, Nong Lam University, Ho Chi Minh City, 700000 Vietnam; 2 Faculty of Agriculture and Food Technology, Tien Giang University, Tien, Giang Province Vietnam; 3 Institute of Applied Technology and Sustainable Development, Nguyen Tat Thanh University, Ho Chi Minh City, Vietnam; 4 Department of Chemistry, Soongsil University, Seoul, 06978 South Korea

**Keywords:** VD20 broken rice, Pasteurization, Homogenization, Antioxidant acitivity, Aerobic microorganisms, Biochemistry, Chemical biology

## Abstract

The preservation and stability of rice milk products are critical for their commercialization. This study focuses on the effects of homogenization and pasteurization on the stability and microbial safety of rice milk produced from VD20 broken rice, a variety cultivated in Go Cong, Tien Giang, Vietnam. Experiments were conducted by homogenizing the rice milk at four rotational speeds (6000, 8000, 10,000, and 12,000 rpm) for varying durations (5, 10, 15, and 20 min) and pasteurizing temperatures ranging from 80 °C to 95 °C. Homogenization was performed using an IKA T50 ULTRA-TURRAX^®^ homogenizer, and microbial counts were determined using standard plate count methods. The optimal processing conditions were identified as homogenization at 10,000 rpm for 15 min and pasteurization at 90 °C for 15 min, which ensured microbial safety (< 10⁵ CFU/mL) while preserving antioxidant activity (DPPH: 42.35 mgAAE/mL, ABTS: 39.01 mgAAE/mL) and polyphenol content (TPC: 78.55 mgGAE/mL). These findings contribute to optimizing the production and extending the shelf life of rice milk products, thereby enhancing the value of broken rice by-products and supporting the diversification of rice-derived functional beverages.

## Introduction

Rice (*Oryza sativa* L.) is a staple food for many countries worldwide. It is derived from rice kernels through a milling process that removes the husk, with carbohydrates content accounting for approximately 80% of the grain weight. A significant portion of this is in the form of starch, with 100 g of rice containing 75.9 g of carbohydrates, contributing to 90% of the energy provided by rice^1,2^. As such, rice serves as a major source of energy for the human body. Additionally, rice is naturally gluten-free, making it an excellent carbohydrate source for individuals with gluten allergies^3^.

Vietnam is one of the world’s largest rice producers, with an annual production of around 26 to 28 million metric tons (according to the Ministry of Industry and Trade, 2020). Of this, 6 to 6.5 million metric tons are exported annually, while the remainder is consumed domestically. The Mekong Delta is one of the two most significant rice-producing regions in Vietnam, with many provinces relying on agricultural economies. Among them, Tien Giang is a key contributor, producing over 1.1 million metric tons of rice annually. A prominent variety cultivated in this region is VD20 rice, a fragrant, short-term rice variety that thrives in the nutrient-rich, silted lands of Go Cong Tay in Tien Giang^4^. VD20 rice is favored for its high adaptability and resistance to pests and diseases, yielding 3 to 4 metric tons/ha in the summer-autumn crop and 4 to 5 metric tons/ha in the winter-spring crop^5^. Over the years, a specialized cultivation area of nearly 4,500 hectares has been established for VD20 rice, accounting for more than 50% of the region’s planting area.

Despite the significant cultivation and availability of rice, rice-based beverages remain underdeveloped in the consumer market. These beverages, made entirely from natural ingredients, align with the modern trend of consumer products derived from nature. In addition to using whole grain rice, the by-products of the milling process, such as broken rice grains, remain largely underutilized. Although broken rice holds equivalent nutritional value to whole grain rice, it is often discarded or sold at a lower price^6^. This presents a unique opportunity to create valuable products from broken rice, potentially improving profits, expanding the market for rice-based products, and increasing the variety of nutritious products available from natural sources.

Research on the development of rice-based products, particularly utilizing broken rice, has gained increasing attention due to its potential to enhance the value of by-products and provide economic benefits to farmers. Studies have demonstrated the versatility of rice milk as a key product in this context. For example, fermented rice milk has been explored as a coagulant in paneer (soft cheese) production, where it positively influenced the physicochemical properties, structure, and shelf life of the final product^7^. Additionally, fermented rice milk beverages containing probiotics and fruit flavors have been developed, showing improved solid, fat, and ash content, as well as enhanced organoleptic and antioxidant qualities^8^. Despite the variety of rice-based products available, rice milk stands out due to its nutritional value, versatility, and growing consumer demand for plant-based beverages. However, limited research has focused on leveraging broken rice, particularly from the VD20 variety, to produce rice milk, presenting a significant opportunity to transform this by-product into a valuable and nutritious commodity.

Previous studies have primarily focused on developing the production processes for rice milk and related products without fully addressing shelf-life extension. To ensure the quality and safety of rice milk products, eliminating bacteria and pathogenic microorganisms while extending the product’s shelf life is essential^9^. Pasteurization, a heat treatment process, is widely used to inhibit the growth of microorganisms and prolong the shelf life of various food products, especially canned goods^10^. It is a crucial step in food preservation, as it inactivates enzymes and inhibits microflora, thereby prolonging the shelf life of the product without compromising taste or nutritional properties^11,12^.

Numerous studies have demonstrated the effectiveness of pasteurization in extending product shelf life. For instance, a study^13^ investigated the impact of sterilization on the quality of water milk, finding that pasteurization at 121 °C for 10 min optimized sensory characteristics, ensured food safety, and extended shelf life. Similarly, other research^14,15^ explored the effects of enzyme treatments and pasteurization on juice quality, revealing that pasteurization at 95 °C for 10 min successfully inhibited microbial growth while preserving the product’s quality.

Thus, pasteurization is a widely accepted method for extending product shelf life due to its effectiveness in reducing microbial load and maintaining product quality. Furthermore, rice milk, an oil-in-water emulsion, tends to separate during storage, necessitating homogenization to enhance product stability. Pasteurization, alongside homogenization, is a crucial step in the production of stable and safe rice milk products. This study aims to evaluate both pasteurization and homogenization processes, combining physico-chemical, biological, and microbiological analyses to develop an optimal rice milk formula from broken VD20 rice. By doing so, the research aims to explore the diversification of broken rice application and creating high-value, and create high-value, nutritious products that can potentially contribute to the sustainable development of local agricultural economies.

## Materials and methods

### Materials, chemicals and equipment

#### Ingredients

**Fig. 1:**
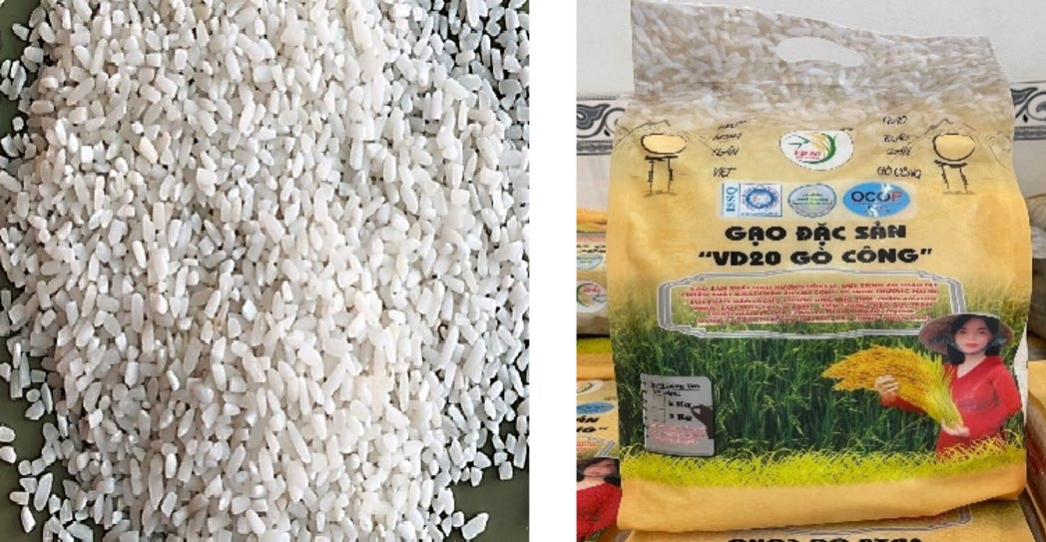
Image of broken rice VD20.

VD20 broken rice was supplied by HK Green Production Co., Ltd., My Tho City, Tien Giang, Vietnam. The rice was clean, free from impurities, with an ivory-white color, a moisture content of less than 10%. (Fig. 1)

#### Chemicals

The chemicals used in this study included α-amylase (99%, 100,000 U/mL) and gluco-amylase (250,000 U/mL) from AngelYeast Co., Ltd. (China); Folin-Ciocalteu reagent (99%), DPPH (99%), and ABTS (98%) from Sigma Aldrich (Germany); KH₂PO₄ (99.5%), NaCl (99.5%), AlCl₃ (97%), Na₂HPO₄·12 H₂O (99%), and peptone from animal tissue from Xilong Scientific Co., Ltd. (China); and ethanol (96%) and CH₃COOK (99%) from Vietnam.

#### Equipment

The key equipment included a thermostat tank (LB-WD316, LKLAB, Korea); an IKA T50 ULTRA-TURRAX^®^ homogenizer (IKA, Germany); a Brookfield viscometer (Brookfield, USA); and a Cary 60 UV-Vis spectrophotometer (Agilent, USA).

### Production process of broken rice milk

The production of rice milk from broken VD20 rice involved several critical steps. Firstly, VD20 broken rice from Go Cong was washed thoroughly to remove impurities and then roasted at 200 °C for 15 min to enhance its color and flavor. The roasted rice was finely ground into a powder and gelatinized in a thermostat tank at 95 °C for 15 min with a water-to-rice ratio of 10:1. Next, enzyme liquefaction was performed to break down long-chain carbohydrates into shorter molecules using α-amylase (100 U/g) at 95 °C for 30 min. The liquefied solution then underwent saccharification by adding gluco-amylase (200 U/g) at 65 °C for 2 h, which hydrolyzed the short-chain carbohydrates into simple sugars.

The saccharified rice solution was filtered through a 10 μm cloth to remove residues. The filtered solution was then blended with stabilizers, including 1.5% sunflower oil and 1.5% whey protein powder, to enhance its nutritional profile and stability. Homogenization was conducted using an IKA T50 ULTRA-TURRAX^®^ homogenizer at various rotational speeds (6000, 8000, 10000, and 12000 rpm), corresponding to approximate G-forces of 32.2 g, 57.3 g, 89.5 g, and 129 g, respectively (calculated for a rotor radius of 0.1 m). The homogenization process was carried out for durations of 5, 10, 15, and 20 min, as per the experimental design.

After homogenization, the rice milk was filled into 100 mL glass bottles and sealed. The bottles were then pasteurized at different temperatures (80 °C, 85 °C, 90 °C, and 95 °C) for varying durations (5, 10, 15, and 20 min). Post-pasteurization, the samples were cooled to 4 °C^16^ and stored for subsequent analyses, including evaluations of color changes, microbial counts, and bioactive compound content.

### Methods for analyzing indicators

#### Total aerobic microorganisms (CFU/mL)

Microbial counts were determined by culturing samples on plate count ager under aerobic conditions at 35 °C for 48 h. The number of colonies per mL was calculated and compared with the permissible standards for liquid milk products (total aerobic microorganisms < 5 × 10⁵ CFU/mL) as per Ministry of Health Regulation 46/BYT and Circular No. 35/2010/TT-BYT^17–19^.

#### Total polyphenol content (mg gallic acid equivalent (GAE)/mL)

The Folin-Ciocalteu method was employed to quantify polyphenols^20,21^. A 10 mL sample of rice milk was diluted to 50 mL with ethanol, filtered, and mixed with 1 mL of Folin-Ciocalteu reagent, 0.8 mL of 7.5% Na₂CO₃, and incubated for 1 h. The absorbance was measured at 765 nm using a UV-Vis spectrophotometer, and the total polyphenol content was calculated based on a gallic acid calibration curve.

#### Total flavonoid content (mg quercetin equivalent (QE)/mL)

Flavonoid content was determined using the AlCl₃ colorimetric method. A 10 mL sample was diluted to 50 mL with ethanol, filtered, and mixed with 4.3 mL of ethanol, 0.1 mL of 1 M CH₃COOK, and incubated for 30 min. Absorbance was measured at 415 nm on a UV-Vis spectrophotometer^22,23^.

#### Antioxidant capacity according to DPPH (mg Ascorbic Acid Equivalent (mgAAE)/mL) and ABTS (mgAAE/mL) radical Scavenging acitivity

The antioxidant capacity was measured based on the ability to neutralize DPPH radicals, which form DPPH-H reduction products. This reaction turns the solution from purple to yellow-orange, reducing its absorbance at 517 nm. Prepare 10 mL of sample to 50 ml with alcohol and proceed to filtration, sucking 0.5 mL of filtrate, 1.5 mL of standard DDPH and incubating for 30 min, respectively. Optical absorption was measured at a wavelength of 517 nm on a UV–Vis spectrophotometer, with the white sample being alcohol^18,24^.

ABTS^+^[2,2’-azinobis (3-ethylbenzothiazoline-6-sulfonate)] is a stable free radical that emits blue fluorescence, with a characteristic absorption wavelength of 734 nm^25^. When an antioxidant compound is added, ABTS^+^ is reduced to colorless, resulting in reduced characteristic wavelength absorption. Antioxidant activity was assessed in vitro through ABTS^+^ free radical fishing. When encountering a solution containing ABTS^+^, the antioxidants will deionize this to ABTS. The oxidation resistance is shown by the neutralizable ABTS content of the test specimen. The higher the ABTS content, the better the oxidation resistance of the test specimen. Prepare 10 mL of sample to 50 mL with alcohol and proceed to filtration, suck 0.5 ml of dialysate, 1.5 mL of standard ABTS and incubate for 30 min, respectively. Optical absorption is measured at a wavelength of 734 nm on a UV–Vis spectrophotometer, with the white sample being alcohol.

#### Viscosity (cP)

Viscosity was measured using a Brookfield viscometer at 100 rpm. After pasteurization, the viscosity of each sample was measured using an LV-51 probe, and the results were recorded after 2 min of stabilization. Each measurement was repeated three times for accuracy.

#### Color value

The color of the rice milk samples was measured using the CIE Lab* color space, with L* representing brightness (0 = black, 100 = white), a* representing green to red, and b* representing blue to yellow. Color measurements were taken three times at different positions on each sample using a colorimeter^26^.

#### Stability determination methods

The stability was evaluated by measuring the height of the separated layer in relation to the total liquid volume after the product had been stored at cooler temperatures for 24 h, following the procedure outlined by Tan et al.^27^. A 10 mL sample of the product was kept under cooler conditions, and the height of the separate layer was recorded after 24 h.

### Statistical analysis

Data were processed using Microsoft Excel^®^ 2016, and statistical analyses were performed using ANOVA and LSD tests to compare the effects of the various factors. The analyses were conducted with a 95% confidence level using Statgraphics Centurion XV software (Statgraphics Technologies, Inc., USA). Each treatment was replicated three times, and each analysis was performed in triplicate to ensure statistical reliability.

## Results and discussion

### Effect of homogenization on the stability of broken rice milk products

Rice milk is an oil-in-water emulsion system, which is inherently susceptible to phase separation due to the collision and coalescence of fat globules. This phenomenon can lead to the formation of larger particles, thereby reducing the product’s stability and negatively affecting its sensory qualities^28^. Consequently, selecting optimal homogenization parameters is essential to reduce the size of the dispersed particles and minimize phase separation, ultimately enhancing the overall stability of the product. The percentage of phase separation (sedimentation) is a key indicator in determining the optimal homogenization conditions, where a lower phase separation percentage indicates a more stable emulsion system^29,30^.

**Fig. 2:**
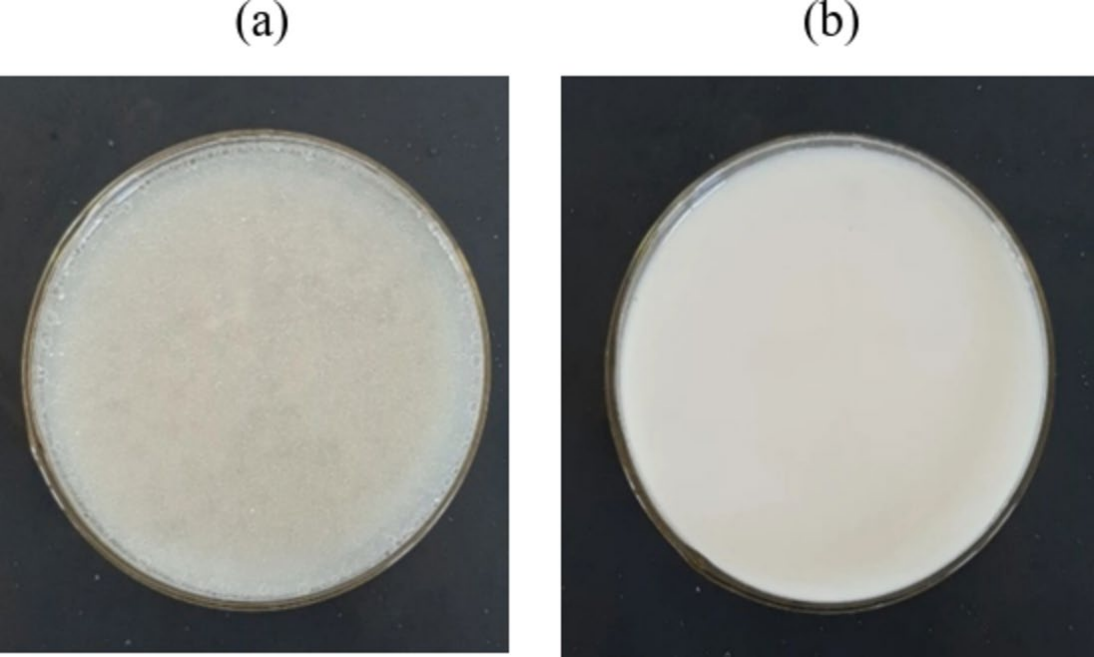
The change of nutritious rice milk products (a) before and (b) after homogenization.

As shown in Fig. 2, there is a noticeable transformation in the consistency of rice milk before and after homogenization. Before homogenization, the rice milk contains a significant number of suspended fat globules that are relatively large and unevenly distributed. However, after the homogenization process, the size of the fat globules decreases significantly, and the dispersed phase (fat globules) becomes smaller and more uniformly distributed within the continuous phase. The product acquires a smoother, more homogeneous texture, which is visually apparent. Thus, it is evident that the homogenization process improves product uniformity and enhances its stability. To evaluate this effect, rice milk products were mixed with additives, homogenized at the selected parameters, with 10 ml samples extracted into test tubes. These samples were then refrigerated, and the percentage of phase separation was measured after 24 h.

**Fig. 3:**
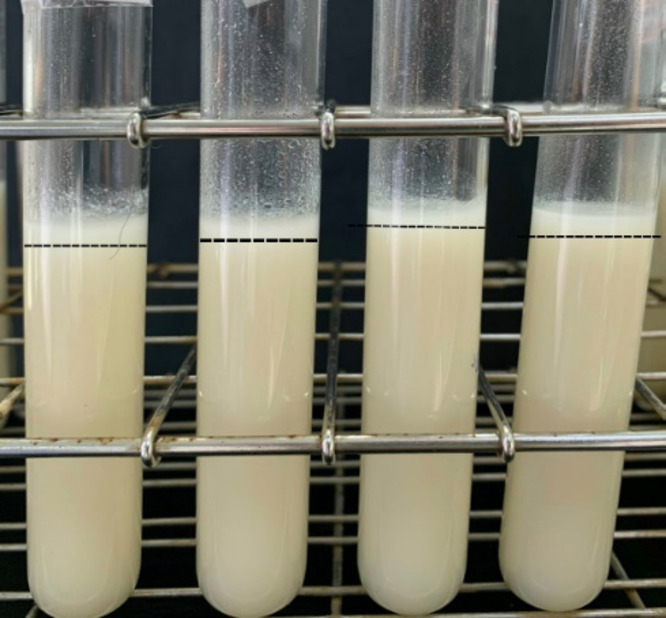
The phase separation of rice milk after 24 h at an homogenization rate of 10,000 rpm.

Figure 3 illustrates the variation in phase separation of rice milk solutions stored at refrigeration temperature for 24 h, as influenced by different homogenization times. At a constant homogenization rate, the sample processed for 5 min exhibited the highest degree of phase separation. This gradually decreased, reaching the lowest level at 15 min. After 20 min, phase separation began to increase slightly again. This pattern highlights the importance of identifying the optimal homogenization time to achieve maximum product stability.

**Fig. 4:**
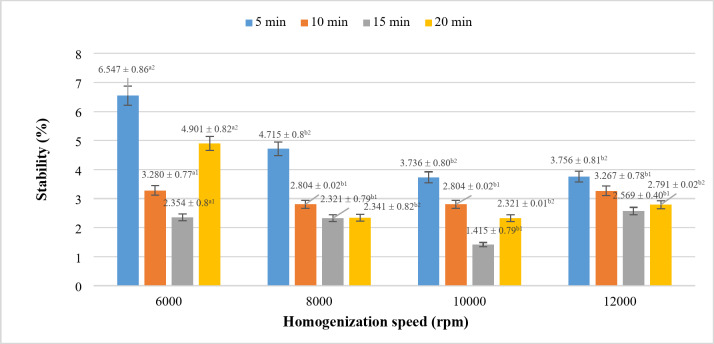
Effect of homogenization on the stability of rice milk after 24 h. *where (a-d) indicates a statistically significant difference (*p* < 0.05).

The results presented in Fig. 4 demonstrate that with increasing rotational speed and prolonged homogenization time, the extent of phase separation initially decreases and then exhibits a slight increase. Specifically, with regard to rotational speed, phase separation decreases progressively from 6000 rpm to 10,000 rpm, followed by a minor increase at 12,000 rpm. The most significant phase separation occurs at 6000 rpm, showing a statistically significant difference compared to the other conditions. This could be attributed to the insufficient energy and interaction force at lower speeds, which are unable to disrupt the intermolecular bonds effectively. As the rotational speed increases, the fat particles become more finely dispersed, reducing their size and distributing them more uniformly in the continuous phase^31^. However, when the speed reaches 12,000 rpm, it exceeds the dispersion limit for the fat droplets, leading to coalescence and the formation of larger particles. This reaggregation causes phase separation to increase, resulting in reduced product stability^32,33^.

In terms of time, the highest phase separation is observed at 5 min across all temperature conditions, likely due to insufficient time to break the bonds between fat globules. As homogenization time increases, phase separation decreases, reaching a minimum at 15 min, particularly at 10.000 rpm – the condition with the least phase separation compared to all other tested parameters. Beyond 15 min, at 20 min, phase separation begins to increase again. This phenomenon parallels the effect of excessive rotational speed, where prolonged homogenization time surpasses the optimal threshold, leading to increased phase separation^34^.

In conclusion, homogenization improves the stability of the emulsion system, minimizing phase separation. However, excessive rotational speed and extended homogenization time can compromise product stability. Therefore, it is essential to determine the appropriate parameters to limit phase separation and maintain product stability. The findings indicate that 10.000 rpm for 15 min provides the most stable product, with the least phase separation, making it the optimal condition for producing stable rice milk products.

### Effect of pasteurization mode on the ability to kill microorganisms of broken rice milk products

The primary objective of pasteurization is to inhibit microbial growth and inactivate spoilage enzymes. According to Vietnam’s Ministry of Health Regulation 46/BYT and Circular No. 35/2010/TT-BYT, the permissible level of total aerobic microorganisms in liquid milk products is < 5 × 10⁵ CFU/mL. Thus, determining appropriate pasteurization parameters is essential for ensuring food safety in broken rice milk products.

**Table 1:** Test results for total aerobic microorganisms (CFU/mL) on pasteurization of rice milk.

Time (min)	Pasteurization temperature (^o^C)			
	80	85	90	95
5	4.2 × 10^3^	3.7 × 10^3^	1.9 × 10^3^	1.0 × 10^2^
10	3.9 × 10^3^	3.2 × 10^3^	1.2 × 10^3^	< 10^2^
15	3.4 × 10^3^	2.8 × 10^3^	5.4 × 10^2^	-
20	2.5 × 10^3^	2.1 × 10^3^	3.2 × 10^2^	-

- : not detected. Results were expressed as less than 10 CFU/g or less than 1 CFU/mL for liquid samples when no colonies were observed on the plate.

Table 1 shows that ensuring product safety is a key criterion in food processing, with the total aerobic microorganism count at all four time and temperature milestones remaining within the permissible limit (< 5 × 10⁵ CFU/mL). As pasteurization conditions change, the microorganism count tends to decrease with increasing time and temperature. The highest total microorganism count was observed at 80 °C for 5 min, likely due to the relatively low temperature and short pasteurization duration, which provided insufficient energy to effectively eliminate all microorganisms.

In contrast, as the pasteurization temperature increased to 90 °C for 15 min, a notable reduction in microbial content was observed. This decrease became even more significant at 95 °C for 15 and 20 min, where the microorganism count dropped to undetectable levels. These results are consistent with similar studies, such as research by Klaudia et al.^35^, and Sethi et al.^36^, which also demonstrated that increasing pasteurization temperature and time effectively reduced microbial counts in dairy and plant-based milk products. For example, Pham et al., 2024 ^27^ found that pasteurization at 90 °C for 10 min resulted in a significant reduction of microbial load in beverages product from VD20 broken rice, similar to the findings in our study for broken rice milk.

These results indicate that pasteurization time is a critical factor influencing the total aerobic microorganism count. Longer pasteurization periods result in a lower number of aerobic microorganisms, thereby enhancing product safety. Consequently, a temperature range of 90 °C for 15 min to 95 °C for 20 min is recommended as an optimal condition for ensuring microbial safety in food products, aligning with the findings of Khan et al.^37^ who also recommended similar conditions for pasteurizing plant-based milk alternatives.

### Effect of pasteurization mode on TPC

Phenolic compounds represent one of the most significant constituents in plants, comprising a substantial proportion of their composition. These compounds are recognized for their potent antioxidant properties, making them a critical indicator in studies of the antioxidant activity of plant-based materials.

**Fig. 5:**
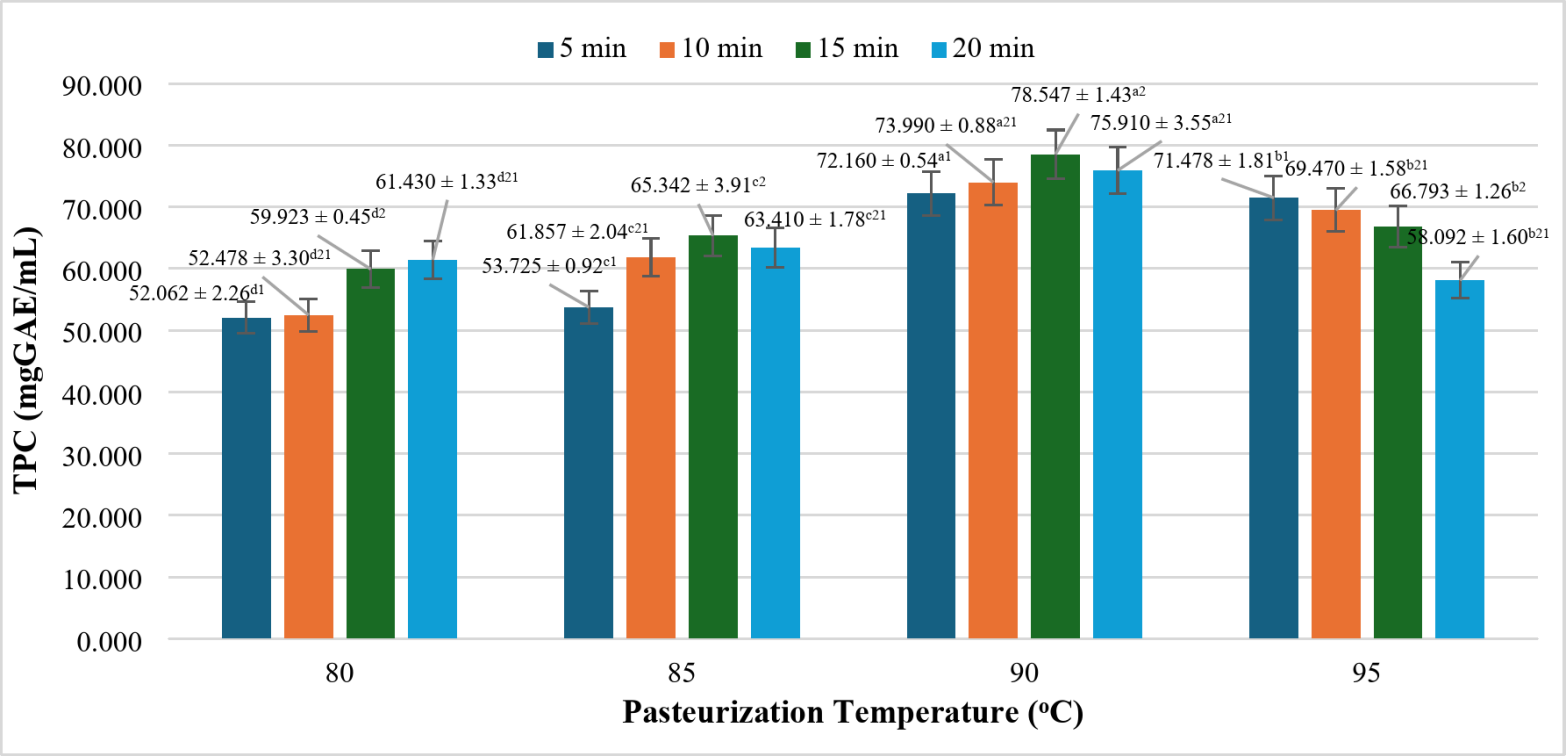
Effect of pasteurization on TPC content in rice milk. *where (a-d) indicates a statistically significant difference (*p* < 0.05).

As illustrated in Fig. 5, the TPC of the samples initially increases with rising temperature and pasteurization time, before exhibiting a subsequent decline. Specifically, the TPC gradually increases from 80 °C to 90 °C, followed by a decrease at 95 °C. This trend can be attributed to the high temperature, which facilitates the release of phenolic compounds from their bound forms, leading to the conversion of insoluble phenolic compounds into soluble forms^38,39^. Additionally, the breakdown of lignin occurs, resulting in the release of phenolic acid derivatives and potentially the formation of new phenolic compounds^40^. Similarly, the impact of pasteurization time is observed, with TPC rising from 5 min to 15 min before decreasing slightly at 20 min. The TPC of the rice milk samples begins to rise gradually, starting from 80 °C for 5 min (52.062 ± 2.26 mg GAE/mL), reaching its peak at 90 °C for 15 min (78.547 ± 1.43 mg GAE/mL). However, at the 20-minute mark at 90 °C, the TPC declines slightly to 75.910 ± 3.55 mg GAE/mL, a change that is not statistically significant (*p*> 0.05). The decrease in TPC at elevated temperatures and extended pasteurization times may be due to the thermal transformation of polyphenolic compounds (particularly from 90 °C at 20 min or longer)^18,27^.

The observed increase in TPC aligns with findings from a study on Ha Chau strawberry juice conducted by^41^, which reported that polyphenol content rises as temperature increases to 85 °C (258.56 mg GAE/mL) but declines slightly beyond this point, with the decline being statistically insignificant. Furthermore, the statistical analysis using ANOVA confirmed a significant influence of time and temperature on TPC (*p* < 0.05). Phenolic compounds in strawberry juice are predominantly flavonoids (e.g., anthocyanins, quercetin) and ellagitannins, which contribute to its antioxidant properties and distinctive color. The TPC reached its highest value at 90 °C for 15 min (78.547 ± 1.43 mg GAE/mL. Therefore, this specific heat-time combination can be considered optimal for maximizing TPC in rice milk products.

### Effect of pasteurization mode on TFC

**Fig. 6:**
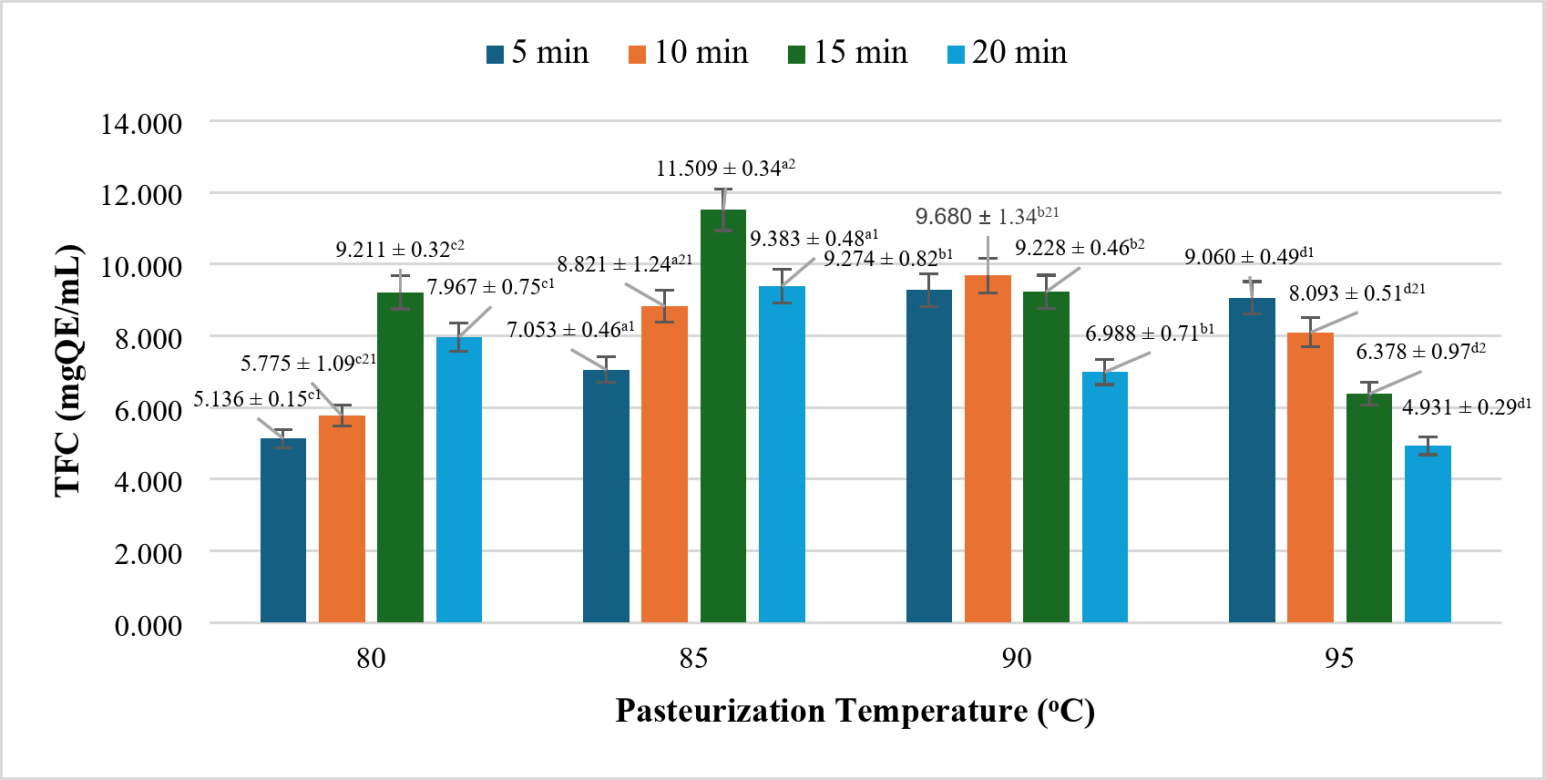
Effect of pasteurization on TFC content in rice milk. *where (a-d) indicates a statistically significant difference (*p* < 0.05).

Flavonoids, like polyphenols, are sensitive to heat treatment. As shown in Fig. 6, it is evident that when evaluated at the same pasteurization temperature, flavonoid compound is more rapidly degraded compared to the polyphenol compound. Specifically, the TFC rises to 11.509 ± 0.34 mg QE/mL at 85 °C for 15 min, followed by a slight decrease to 9.383 ± 0.48 mg QE/mL at 85 °C for 20 min, with further reductions observed at subsequent time points. The peak TFC occurs at 85 °C for 15 min, with statistically significant variations across different temperatures and heating durations. This rapid decline can be attributed to the sensitivity of flavonoids in aqueous solutions, which varies based on the chemical structure of the compound; however, prolonged thermal exposure at elevated temperatures gradually degrades this structure^42^. Consequently, the optimal pasteurization parameters for preserving TFC can be identified as 85 °C for 15 min, under which conditions the TFC reaches its maximum value.

In summary, both total polyphenol content and total flavonoid content are temperature-sensitive components. Generally, heat treatments, including pasteurization, result in modifications to phenolic compounds^43,44^. Notably, there are exceptions, such as in the case of apple juice, where increasing the processing temperature from 40 °C to 70 °C results in a 50% increase in flavonoid content^45^, and similar increases in tomato flavonoids during heat treatment have also been documented (Dewanto et al., 2002). Therefore, it is essential to identify appropriate pasteurization parameters to optimize these two components.

For nutritious rice milk products, the study identified two suitable parameters: 90 °C for 15 min and 85 °C for 15 min. Further evaluation of additional criteria such as oxidative stability, viscosity, and color is necessary to determine the most optimal pasteurization conditions for the final product.

### Effect of pasteurization mode on oxidation resistance according to DPPH

The antioxidant activity of nutritious rice milk is primarily reflected in its capacity to capture free radicals, specifically through DPPH and ABTS assays. This property serves as a critical indicator for assessing the health benefits of food products. Numerous studies indicate that both temperature and duration of processing are significant factors influencing antioxidant activity. Variations in temperature can modify the mechanisms of action of certain antioxidants or affect their stability^46^.

**Fig. 7:**
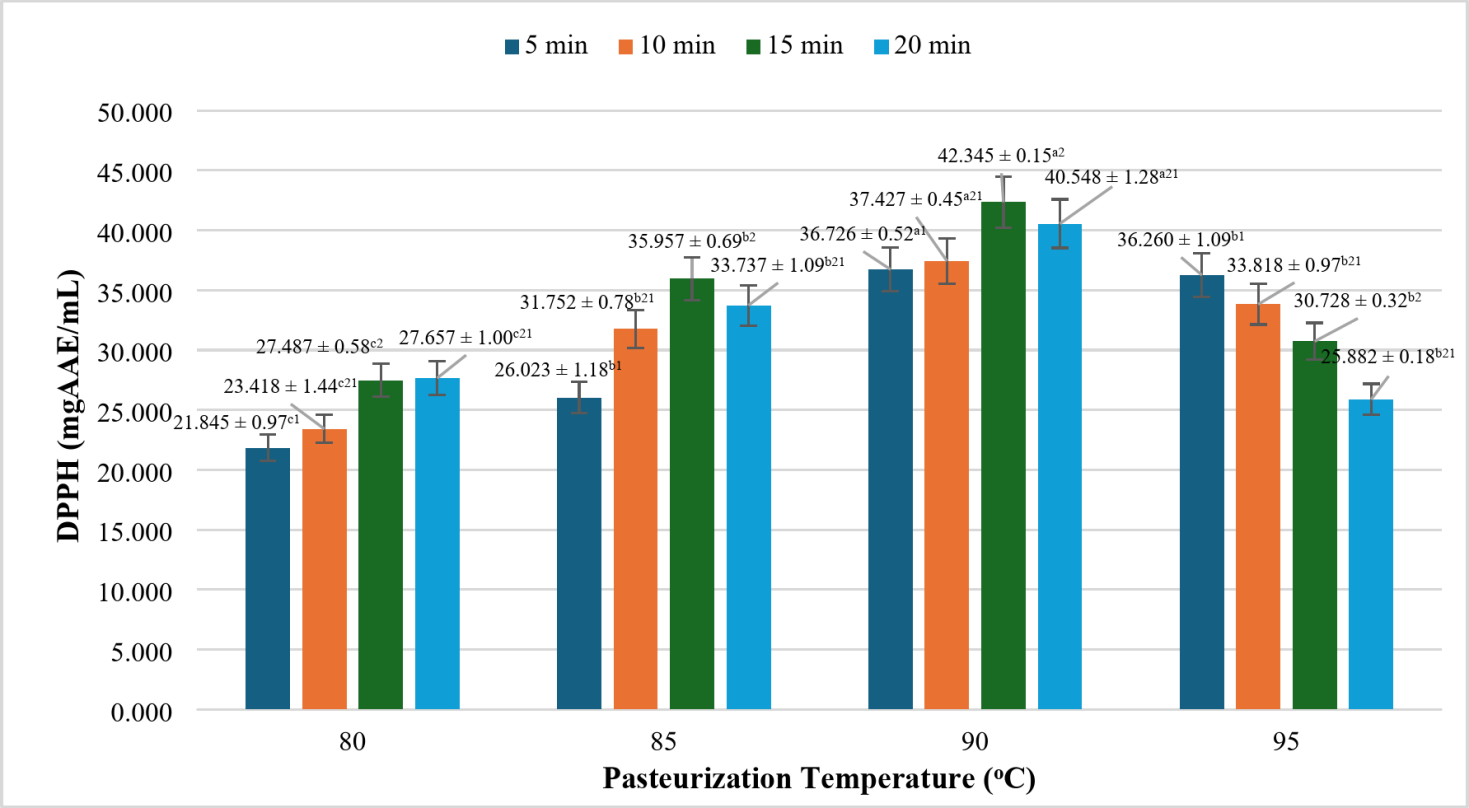
Effect on pasteurization on DPPH free radical capture (mgAAE/mL). *where (a-d) indicates a statistically significant difference (*p* < 0.05).

As depicted in Fig. 7, the ability of rice milk products to scavenge free radicals, as measured by the DPPH assay, generally increases up to a specific threshold of temperature and time before gradually declining. More specifically, the capacity to capture free radicals rises steadily from 80 °C for 5 min to a peak at 90 °C for 15 min, after which a gradual decrease is observed. This enhancement in antioxidant activity correlates with an increase in the concentrations of total polyphenols and flavonoids, which are influenced by thermal interactions and processing time^46^. The modest growth in antioxidant activity at lower temperature intervals is attributed to insufficient thermal energy to hydrolyze the pre-active compounds present in the product^47^.

At the pasteurization temperature of 90 °C for 15 min, the antioxidant activity reaches its highest value of 42.345 ± 0.15 mg AAE/mL, which is statistically distinct from the other conditions assessed. However, when the temperature is increased to 95 °C, the molecular structure of less heat-stable antioxidants may be altered. According to We et al.^48^ and Polisjak et al.^49^, heat can lead to the evaporation of certain antioxidants, resulting in the loss of biological activity and a subsequent reduction in antioxidant efficacy compared to lower temperature and shorter duration pasteurization methods.

These findings underscore the significant impact of temperature and pasteurization time on the antioxidant activity of active components in nutritious rice milk products. The optimal pasteurization parameters identified, specifically 90 °C for 15 min, can be considered suitable for maximizing oxidation resistance in the final product.

### Effect of pasteurization mode on oxidation resistance according to ABTS

**Fig. 8:**
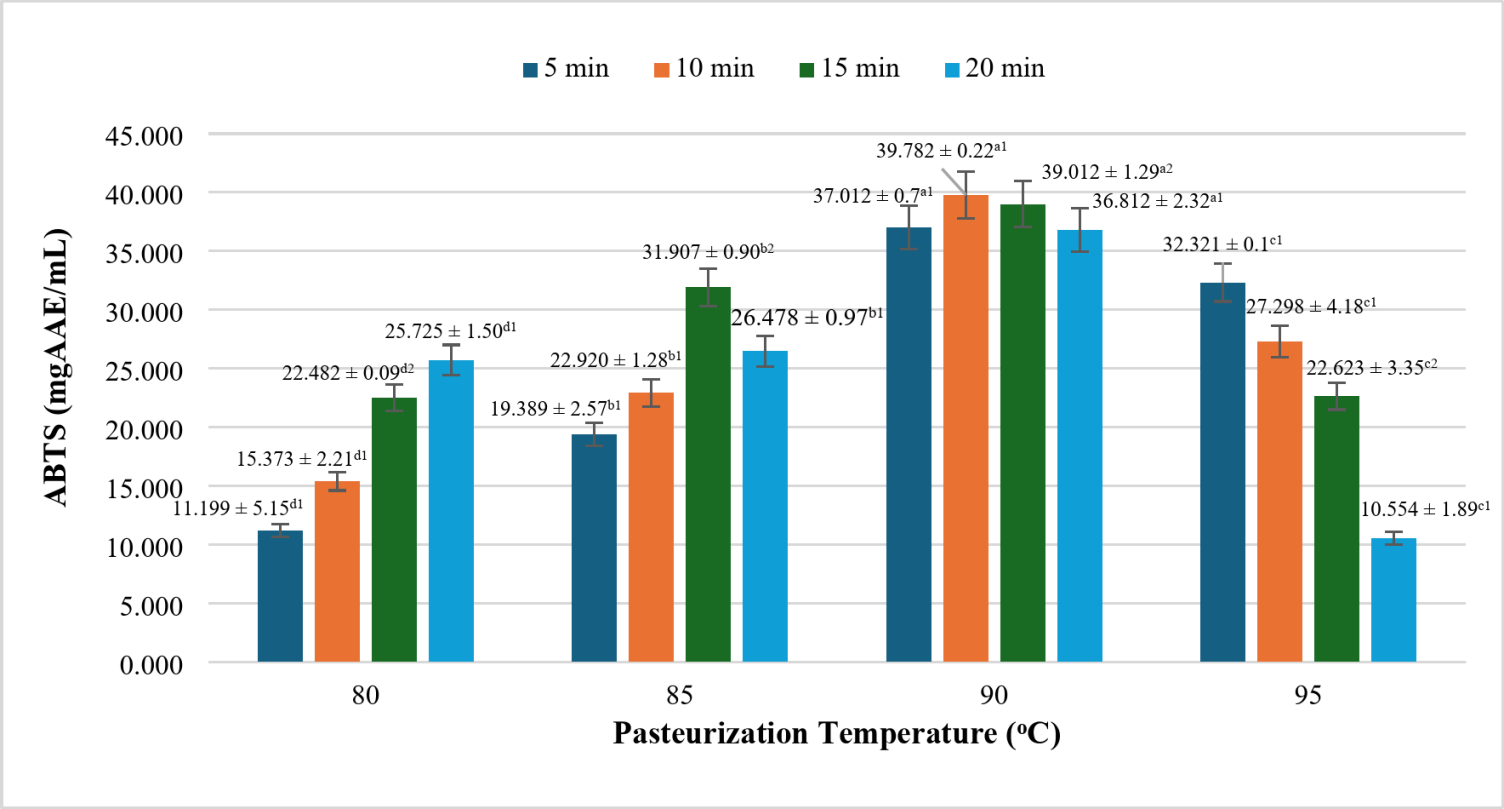
Effect on pasteurization on ABTS free radical capture (mgAAE/mL). * Where (a-c) and (1–2) indicate the statistically significant difference (*P* < 0.05).

Similar to the DDPH results, the oxidation resistance of rice milk products, as measured by the ABTS assay, demonstrates a gradual increase with rising temperature and pasteurization time, followed by a sharp decline at the temperature threshold of 95 °C (Fig. 8). Specifically, the product’s capacity to capture free radicals via the ABTS assay exhibits a significant increase at 90 °C, reaching its peak value of 39.782 ± 0.22 mg AAE/mL at 90 °C for 10 min. Notably, there are no significant differences in antioxidant activity among various time points when the pasteurization temperature is maintained at 90 °C, suggesting that this temperature provides stability in antioxidant activity compared to other thermal conditions.

The stability observed at this temperature may be attributed to the relatively consistent polyphenol content, which appears to remain more stable at 90 °C than at other temperatures. However, a marked decrease in antioxidant capacity is observed at 95 °C, particularly with extended pasteurization time. The lowest antioxidant activity recorded via the ABTS assay occurs at 95 °C for 20 min, measuring at 10.554 ± 1.89 mg AAE/mL. This decline can be explained similarly to the observed reduction in DPPH scavenging ability; excessive pasteurization at high temperatures likely leads to the decomposition of antioxidant compounds, thereby diminishing their oxidative capacity^50,51^.

### Effect of pasteurization mode on product viscosity

Nutritious rice milk products were subjected to pasteurization at various temperatures (80 °C, 85 °C, 90 °C, 95 °C) and times (5, 10, 15, 20 min), followed by viscosity measurements using a DV1M Brookfield viscometer. Viscosity is a crucial parameter for assessing suspension products, as higher viscosity generally correlates with greater product stability^52^.

**Table 2:** Temperature and pasteurization time influence on product viscosity of rice milk.

Time (min)	Pasteurization temperature (^o^C)			
	80	85	90	95
5	4.32 ± 0.06^a1^	3.90 ± 0.23^b1^	3.49 ± 0.12^c1^	3.26 ± 0.03^d1^
10	4.10 ± 0.11^a2^	3.58 ± 0.07^b2^	3.29 ± 0.08^c2^	3.07 ± 0.02^d2^
15	3.88 ± 0.07^a3^	3.32 ± 0.07^b3^	3.13 ± 0.06^c3^	2.92 ± 0.14^d3^
20	3.65 ± 0.14^a4^	3.14 ± 0.06^b4^	2.99 ± 0.04^c4^	2.67 ± 0.20^d4^

* Unit: cP. Note: The figures in the table are averages of 3 repetitions. In the same column, different letter-followed numbers represent between values that have a difference in statistical significance at *p* < 0.05.

The results indicate that both temperature and pasteurization time significantly influence the viscosity of the product. Specifically, the viscosity of nutritious broken rice milk exhibits a gradual decline with increasing temperature and pasteurization duration. This phenomenon can be attributed to the role of proteins within the product, which serve as protective agents for fat droplets in the emulsion system^53^. However, these proteins are susceptible to modification and degradation at elevated temperatures, negatively impacting the viscosity of the product^54^. Similar trends have been observed in other studies. For instance, Aline et al.^55^ reported a similar decrease in viscosity in plant-based milk when pasteurized at higher temperatures. The reduction in viscosity was attributed to protein denaturation and the breakdown of the emulsion, which is consistent with our findings in broken rice milk.

Statistical analysis using ANOVA confirms that the effects of time and temperature on viscosity are statistically significant (*p* < 0.05), with a confidence level of 95%. Notable differences were observed among the various experimental milestones. As presented in Table 2, viscosity values remain acceptable within the temperature range of 85 °C to 90 °C, ensuring product stability.

### Effect of pasteurization mode on the color of the product

Pasteurization is a method of heat treatment that can significantly affect the color of the product during processing.

**Table 3:** The result of the effect of pasteurization on the L* of rice milk.

Time (min)	Pasteurization temperature (^o^C)			
	80	85	90	95
5	68.96 ± 0.08^a1^	68.89 ± 0.15^a1^	65.49 ± 0.06^b1^	62.49 ± 0.08^c1^
10	68.62 ± 0.48^a2^	68.80 ± 0.15^a2^	65.26 ± 0.35^b2^	61.25 ± 0.07^c2^
15	68.53 ± 0.38^a3^	68.78 ± 0.59^a3^	65.17 ± 0.19^b3^	59.70 ± 0.46^c3^
20	68.42 ± 0.13^a4^	67.89 ± 0.21^a4^	65.20 ± 0.04^b4^	58.78 ± 0.21^c4^

Note: The figures in the table are averages of 3 repetitions. In the same column, different lettered numbers are represented between values with differences in statistical significance at *p* < 0.05.

As shown in Table 3, the L*value (lightness) at various pasteurization temperatures demonstrates a significant difference, decreasing with increasing temperature. Notably, the most pronounced reduction occurs at the higher temperatures of 90 °C and 95 °C. This phenomenon is attributed to the saccharification of rice water, which contains reducing sugars like glucose and maltose. Under elevated temperatures, these components interact with amino acids, leading to the Maillard reaction between amino groups and carboxyl groups, resulting in browning^56,57^. As temperature increases, this reaction intensifies, causing a darker product color^58^. The L*value tends to decrease further with prolonged pasteurization time. As the temperature rises and the duration extends, the increased interaction between chemical components enhances the browning reaction, leading to a gradual loss of whiteness and a greater deviation in color (∆E) compared to the unpasteurized sample^59^.

**Table 4:** The result of the effect of pasteurization mode on color deviation ∆E of rice milk.

Time (min)	Pasteurization temperature (^o^C)			
	80	85	90	95
5	0.74 ± 0.04^d3^	0.53 ± 0.07^c3^	3.90 ± 0.08^b3^	6.82 ± 0.09^a3^
10	0.54 ± 0.47^d2^	0.62 ± 0.11^c2^	4.18 ± 0.30^b2^	8.06 ± 0.10^a2^
15	0.62 ± 0.34^d1^	1.06 ± 0.35^c1^	4.24 ± 0.20^b1^	9.78 ± 0.20^a1^
20	0.74 ± 0.13^d1^	1.62 ± 0.19^c1^	4.38 ± 0.05^b1^	10.49 ± 0.20^a1^

Note: The figures in the table are averages of 3 repetitions. In the same column, different lettered numbers are represented between values with differences in statistical significance at *p* < 0.05.

In contrast to the L* value, the trend of color deviation (*∆E*) increases as pasteurization temperature and time rise (Table 4). This indicates that higher temperatures and longer durations lead to a more pronounced color deviation (darker) compared to the unpasteurized sample, highlighting the effects of the Maillard reaction. Therefore, the color results for nutritious rice milk products show statistically significant differences across various temperature ranges. However, the pasteurization times of 15 min and 20 min do not exhibit meaningful differences, allowing for consideration of the color value ranges (L*,* ∆E*, a*, b*) for the 5–10 min timeline.

In summary, both pasteurization time and temperature impact the biochemical compounds, biological activity, and color of nutritious rice milk products. Based on the results, the optimal parameters for pasteurization are determined to be 90 °C for 15 min. This condition effectively maintains the quality of nutritious rice milk products regarding safety (microorganisms), bioactive content, oxidative stability, and other characteristics such as viscosity and color.

### Effect of temperature and pasteurization time on product stability

**Fig. 9:**
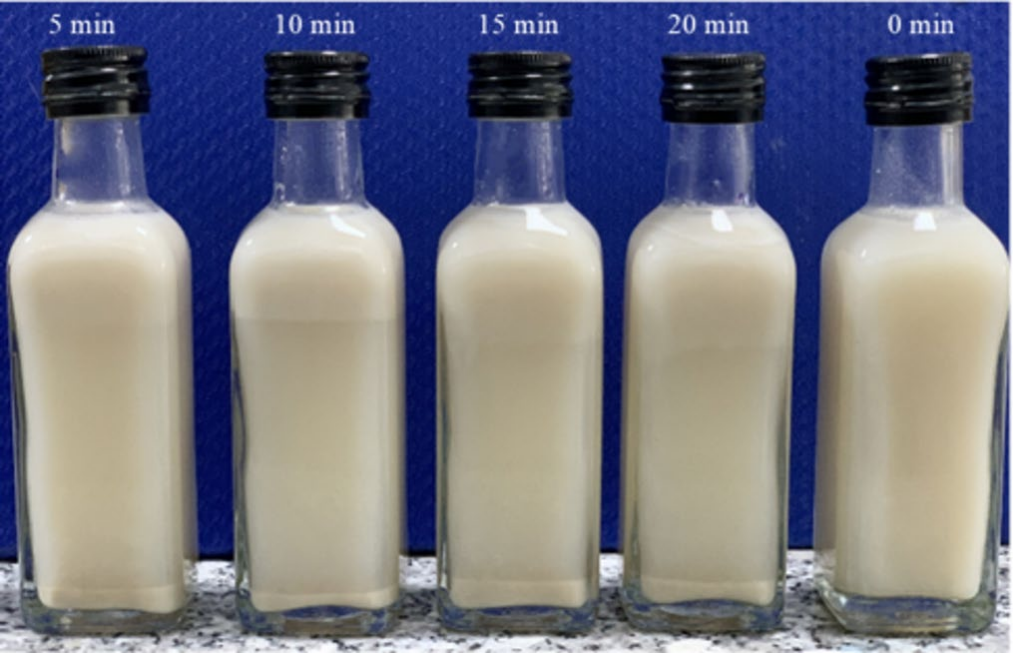
Products after pasteurization through different timelines at 90 ^o^C.

Observing Fig. 9, it is evident that the pasteurized products, across heat retention time milestones of 5 to 20 min, exhibit a thin layer of sediment rising to the surface and a sediment layer accumulating at the bottom of the bottle. The amount of sediment and layering increases progressively from 5 to 20 min, indicating that the pasteurization process significantly impacts the product’s appearance.

The observed changes can be attributed to the effects of temperature and pasteurization time on protein coagulation. As noted in the research by^60^, prolonged pasteurization can lead to the denaturation of heat-unstable proteins within the product. This denaturation causes fat droplets to coalesce, resulting in the flocculation of proteins and contributing to the instability of the product^61^. Such changes become increasingly apparent as the pasteurization time extends from 5 to 20 min.

Thus, it is crucial to select appropriate pasteurization parameters that ensure microbiological safety while retaining biologically active substances and oxidation resistance, all while maintaining acceptable levels of sedimentation. Considering these factors, the parameters of 90 °C for 15 min emerge as optimal, fulfilling all necessary conditions for the quality of the rice milk products.

## Conclusion

The research clearly demonstrates that both the rate and duration of homogenization, as well as the pasteurization conditions (temperature and time), play critical roles in determining the quality of nutritious broken rice milk. Homogenization at 10,000 rpm for 15 min yielded the best stability, minimizing phase separation and ensuring a uniform product. Pasteurization at 90 °C for 15 min was identified as the optimal condition, striking a balance between microbiological safety and the preservation of key physicochemical properties, antioxidant activity, and overall product quality. These findings offer valuable insights for the development of high-quality rice milk with enhanced health benefits, making it suitable for safe and nutritious consumption.

## Data Availability

The datasets used and/or analysed during the current study available from the corresponding author on reasonable request.
